# 

**Published:** 1882-08

**Authors:** 


### CONTENTS.

### COMMUNICATIONS.

Correlated Diseases of the Teeth and Ear.......... 365
Methods and Manipulations with Gold in Filling Teeth.......... 376
Alveolar Abscess.......... 383
Aristocracy of Profession.......... 389
Alimentation.......... 392
Annual Address.......... 402
Dental Diplomas in the State of New York.......... 407
Difficulties Arising from Eruption of a Third Molar.......... 409
Queries.......... 410
Death of Dr. E. M. Nutting.......... 411
The Freezing Cure.......... 411

### EDITORIAL.

Indiana State Dental Society.......... 412
Why Not.......... 414
"I Will Go a Fishing".......... 415
Transactions of American Dental Association.......... 416

